# American Indians and Alaska Natives in the COVID-19 Pandemic: The Grave Burden We Stand to Bear

**DOI:** 10.1089/heq.2021.0011

**Published:** 2021-06-08

**Authors:** Erik Brodt, Allison Empey

**Affiliations:** ^1^ Department of Family Medicine and Oregon Health and Science University, Portland, Oregon, USA.; ^2^ Department of Pediatrics, Oregon Health and Science University, Portland, Oregon, USA.

**Keywords:** COVID-19, health inequities, Native American health, Indigenous health, American Indian health

## Abstract

The United States is bearing witness as a crisis-within-a-crisis unfolds across Indian Country, where a persistently underfunded system with inadequate resources and outdated facilities set the stage for coronavirus disease 2019 (COVID-19) to overwhelm Tribes. Now is the time to reimagine our way forward as a country beyond the pandemic. To address these issues, we recommend that (1) the federal government appropriately fund the Indian Health Service and work more closely with tribal governments, and (2) programs that recruit, train, and retain American Indian and Alaska Native (AIAN) health professionals be expanded. We offer guidance on decisive and impactful steps that can be taken, together, today.

Oregon is a place of stark contrasts, from coastal mountain rainforests to vast basin deserts. The drive to one tribal reservation from Portland winds through old growth forests, skirts Mt. Hood, and drops onto high desert flats of sage brush and wild horses. Working for Oregon Health & Science University (OHSU) Hospital and a tribal health clinic demonstrates an additional painful contrast—stunning health care inequities. The coronavirus disease 2019 (COVID-19) pandemic has laid bare how high rates of poverty, outdated infrastructure, increased prevalence of chronic diseases, and limited access to high-quality health care have the potential to devastate American Indian and Alaska Native (AIAN) people. Simply put, COVID-19 stands to weigh gravely upon Tribes across America.

We are both American Indians, physicians, faculty members, program leaders, educational researchers, and community members—these threads are woven together to afford us a unique perspective on the COVID-19 pandemic and the implications for Indian Country. Make no mistake. We do not pine away dreaming in the ivory tower of academic medicine. We are on the frontlines, caring for patients in both a rural tribal health clinic and attending on COVID-19 services at an academic health center. A crisis-within-a-crisis is unfolding across Indian Country, where persistent structural and systemic inequities set the stage for COVID-19 to devastate Tribes. The current circumstances are preventable and result from a system that is achieving the outcomes of its design—intentional policy decisions, determinations, and breach of contract. Simply put, it does not have to be this way.

We witnessed first-hand the inequitable access to COVID-19 testing that existed in our region at the pandemic's start. Testing at our academic health center was conducted through “respiratory symptom” clinics, drive-thru sites, and hospital-based assays—with no scarcity. The Indian Health Service (IHS) had “access to testing for individuals who may have COVID-19”^1^ with the caveat that regional shortages may exist. For example, one tribal health clinic had capacity to conduct <50 assays for nearly 6000 patients; providers were advised to use outdated testing criteria that included lower respiratory track symptoms, fever, personal contact, or history of travel to China. Yet, one of the first Oregon COVID-19 cases was from community transmission in a rural county with a large Indian reservation.^2^ COVID-19 presence was apparent, as were stark contrasts in care between well-resourced and under-resourced systems. We are deeply troubled by this because to us, Tribes are family and deserve better.

The pandemic is not unfamiliar territory for us. Elders share stories of diseases of the past, and COVID-19 is a sad reminder of those we lost. Millions of our ancestors perished upon the arrival of European settlers and their diseases. Many Tribes adapted and survived—many did not. Responding in the time of COVID-19 is our next life-altering challenge.

The U.S. government signed numerous treaties with Tribes. These treaties are noted in the Constitution as “the supreme law of the land”^3^ and established a unique set of rights, benefits, and conditions to the signing tribe(s) that oftentimes included ceding of millions of acres of homeland in exchange for health care and educational opportunities. The federal trust responsibility to provide health care to Tribes is partially fulfilled through the IHS—an underfunded organization comprising federal, tribal, and urban facilities that serves >2.6 million Natives in 37 states. The IHS had annual expenditures of $4078 per patient in 2017—less than half the national average of $9726.^4^ The average age of an IHS facility is 40 years, compared with 10.6 years nationally. An improperly resourced health system is associated with poor health outcomes. Financial constraints further exacerbate provider shortages and access to care, where one in four postings are vacant in the IHS.^5^ This results in extreme health inequalities for Native people, which are founded and perpetuated by persistent structural and systemic inequities.

Not all will be affected equally by COVID-19. Early evidence suggests that under-represented minority communities, including American Indians and Alaska Natives, experience higher mortality rates from COVID-19 as compared to more advantaged communities.^6–8^ The higher mortality rates are predicated on coexisting health conditions, socioeconomic factors, and barriers to care. Critical illness and mortality from COVID-19 has been correlated with advanced age and coexisting conditions, including diabetes, cardiac disease, kidney disease, obesity, and respiratory diseases.^9^ Native people are highly affected by these coexisting conditions.^10–14^ It is no surprise that Native people are disproportionately affected by the pandemic with 3.5 times the incidence as non-Hispanic whites and the highest hospitalization rate for COVID-19 of any racial or ethnic group.^15,16^ COVID-19 is not “the great equalizer”—COVID-19 is the great illuminator, shining light onto some of the most shameful health inequities in our society.

The potential for profound fatalities due to COVID-19 among Native people is further exacerbated by the underlying social determinants of health—poverty, rural geography, crowded multigenerational housing, and decreased access to safe water—as well as inadequate health services with dated facilities and persistent workforce vacancies. The storm of COVID-19 cases and fatalities on the Navajo Nation is a harrowing forecast of how Native communities stand to be impacted by COVID-19.

Tribal leadership, health services, and epidemiology centers across the country have been preparing with states, the Centers for Disease Control and Prevention, Federal Emergency Management Agency, and other entities to respond to the pandemic, fully anticipating the gravity of the situation. Despite these efforts, planning and preparation will not be enough to overcome funding and other systemic barriers ingrained into the IHS. “We The People” are better than this and must choose a different path.

As a country, we will decide how to move forward after the pandemic. Right now, we must respond by slowing the spread of COVID-19, vaccinating tribal citizens, building tribal public health infrastructure, ensuring access to necessary resources to provide safe and high-quality care, and providing access to daily necessities for survival. When the pandemic passes, we must reimagine our future together, all of us. This reimagined future must include an honest evaluation of the systemic funding, health, and workforce inequities that exist in Indian Country.

The Northwest Native American Center of Excellence (NNACoE) at OHSU is the only Human Resources & Services Administration (HRSA)-funded Center of Excellence (COE) in the country focusing exclusively on training more AIAN physicians. AIAN medical students remain disproportionately under-represented in U.S. medical schools. Few U.S. medical schools train AIANs—more than 40% of medical schools have zero AIAN students enrolled, with 90% of medical schools having three or fewer AIANs.^17^ Training AIAN physicians stands to mitigate both long-term physician vacancies in the Indian health system and reduce the costly financial burden of staffing health facilities with itinerant and locums providers.

In response, NNACoE operates innovative programming at key moments across the educational continuum to increase the number of AIAN physicians. The percentage of AIAN medical students at OHSU is now 3% overall, with 6% AIANs in the Class of 2024. NNACoE is well aware we cannot solely reverse the disproportionate under-representation of AIAN physicians nationally. Now is the time for to increase investments in the HRSA Centers of Excellence and Health Career Opportunity Programs (HCOP), particularly AIAN-specific programs.

The shade has been drawn back on the IHS by COVID-19. It is clear that the federal government must commit to per capita equity and fully fund the IHS. The federal government and tribal governments must continue to work in consultation to resolve needed reforms and alleviate persistent provider vacancies. Health pathway programs (COEs and HCOPs) that train more health professionals to meet the health workforce needs of underserved communities should be expanded. Permanent funding for AIAN-specific HRSA COEs and HCOPs must be mandated and authorized by the federal government. Medical schools must invest time and resources in establishing new relationships and strengthening existing relationships with Tribes. Medical schools must ensure their efforts to develop the future physician workforce includes Native learners and faculty. These structural reforms are critical to ensuring greater health and opportunity to everyone in America.

If you are moved to act, we ask you to do three things. First, support organizations that provide essentials such as food and clean safe water for Native people (First Nations Development Fund & The Navajo Water Project). Second, contact your U.S. Representative and U.S. Senators and compel them to mandate full funding with advanced appropriations for the IHS and permanent funding for AIAN-specific HRSA COEs and HCOPs. Third, challenge the leadership of your regional health professional schools to strengthen relationships with Tribes and commit to programs for Native learners and faculty.

Emerging from the perilous mountain of the pandemic, our country will be indelibly marked by the before and after contrast of COVID-19. This may be the moment when we actualize a future where the status quo of systemic health inequities is no longer tolerated. Beyond the COVID-19 pandemic we must boldly reimagine our way forward, together, as a more healthy and secure country. As American Indian physicians, educators, leaders, and researchers, count us in.

## Abbreviations Used

AIAN: American Indian and Alaska Native
COE: Center of Excellence
COVID-19: coronavirus disease 2019
HCOP: Health Career Opportunity Programs
HIS: Indian Health Service
HRSA: Human Resources & Services Administration
NNACoE: Northwest Native American Center of Excellence
OHSU: Oregon Health & Science University
